# The cytodiagnosis of metastasis of follicular thyroid carcinoma in the scalp: an unusual case report

**DOI:** 10.11604/pamj.2025.50.63.45996

**Published:** 2025-02-27

**Authors:** Shivali Kalode, Arvind Bhake

**Affiliations:** 1 Department of Pathology, Jawaharlal Nehru Medical College, Datta Meghe Institute of Higher Education and Research, Sawangi (Meghe), Wardha, Maharashtra, India

**Keywords:** Fine needle aspiration cytology, follicular thyroid carcinoma, metastasis, scalp, case report

## Abstract

Follicular thyroid carcinoma is known to metastasize to distant places even before the primary follicular thyroid carcinoma is clinically obvious. One such unusual and distant metastasis of follicular thyroid carcinoma is its subcutaneous location. The present case report is an unusual one. A 50-year-old man with a history of trauma on the scalp developed non resolving hematoma. The swelling was soft with greenish discolouration to the skin over it. The swelling was soft with greenish discolouration to the skin over it. The Fine Needle Aspiration Cytology (FNAC) of the swelling revealed the classic cytomorphology of metastatic follicular thyroid carcinoma. The clinical examination of thyroid was normal at this juncture cytodiagnosis. The USG of the thyroid showed a deeply placed nodule of higher Thyroid Imaging Reporting & Data System (TIRAD). The FNAC from this nodule was also performed. The diagnosis of follicular neoplasm favoring follicular thyroid carcinoma was offered. The patient underwent thyroidectomy. The histological examination of tumor tissue was consistent with the diagnosis of thyroid follicular carcinoma. This case offers a unique insight into the non-resolving hematoma harboring the metastatic lesion of follicular thyroid carcinoma in the patient with the silent primary in the thyroid.

## Introduction

The follicular thyroid carcinoma (FTC) is an aggressive thyroid malignancy with high potential [1]. FTC is notorious for producing distant metastasis commonly to lungs and bones apart from lymph nodes in neck [2]. The metastasis of follicular thyroid carcinoma may clinically be appreciated much earlier to the enlargement of the thyroid due to follicular thyroid carcinoma [3]. Follicular thyroid carcinoma has a tendency to invade the blood vessels and therefore hematogenous metastatic spread of it to distant sites is an expected biological phenomenon [4]. Here we report an unusual case diagnosis of fine needle aspiration (FNA) of metastatic cutaneous lesion in the scalp in 50 years old male. This particular patient had a negative lymph node in the neck and locoregional examination of the thyroid was clinically normal.

## Patient and observation

### Patient information

A 50-year-old man reported to the surgery outpatient department with the complaint of swelling over the scalp. The swelling was located at the frontoparital area on the left side of the scalp. The swelling was increasing in size over a period of years. The patient provided a history of fall of recent times. Since then, the swelling was present which on the conventional treatment did not recede. Therefore, he sought the medical opinion.

### Clinical findings

**Figure 1:**
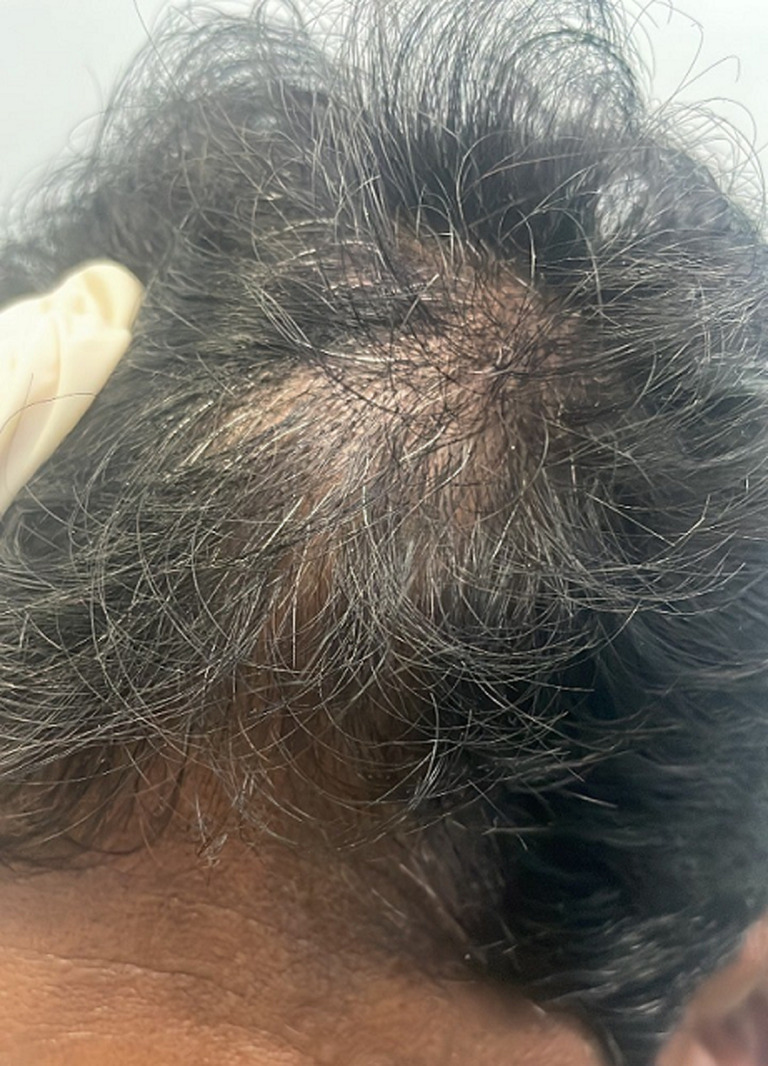
swelling on the scalp on the left side

The patient was examined by the surgeons with a provisional diagnosis of skin adnexal tumor. His systemic examination by the surgeon was reported in normal limits. No lymphadenopathy or any other swelling in the neck or suboccipital region was noticed. His general examination was otherwise normal. The characteristics of the local examination of the swelling were as follows: 1) size - 5 x 3.5 cm, 2) soft non-fluctuant and non-pulsatile, 3) skin over the swelling little greenish hue, well-demarcated swelling, and partly adherent to underlying structure, 4) painless (Figure 1).

### Timeline of the current episode

Swelling over the scalp over a period of years, gradually increasing to the present size.

### Diagnostic assessment

As the swelling was obvious the patient was sent for unguided FNAC in cytopathology OPD. The FNAC was carried out using standard procedure. The aspirate was bloody in character. The smear of the aspirate underwent the staining of May-Grunwald-Giemsa and Papanicolaou stain.

**Figure 2:**
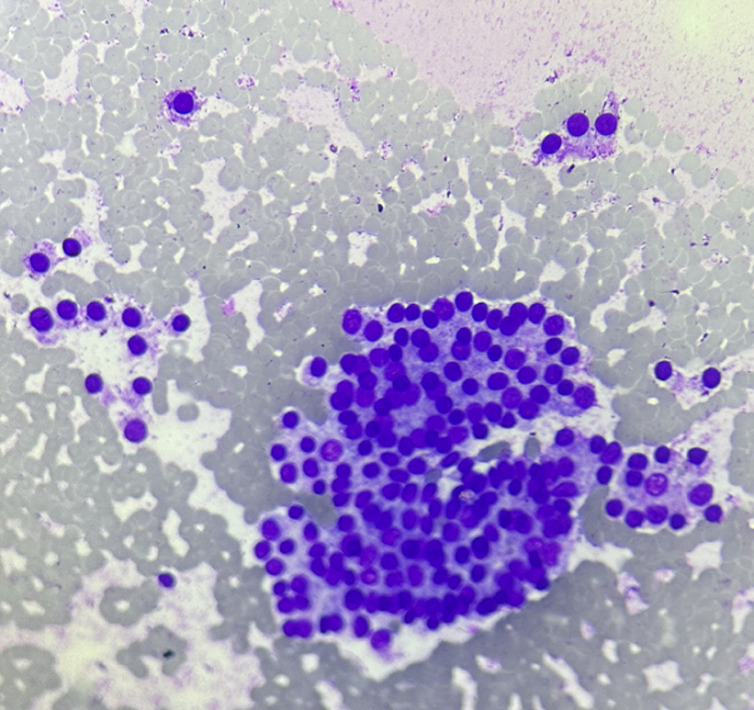
photomicrograph, FNAC scalp swelling, follicular thyroid carcinoma showing multiple microfollicles with nuclear pleomorphism, lack of colloid in the lumen of follicles (10X, MGG)

**Figure 3:**
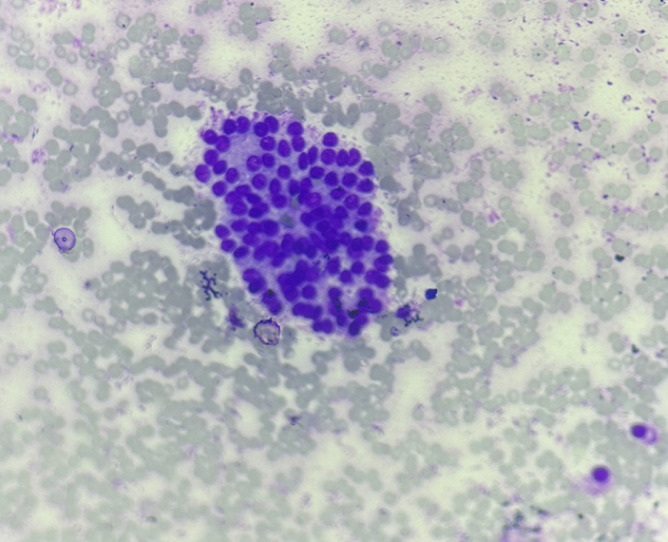
photomicrograph, FNAC scalp swelling, follicular thyroid carcinoma showing sheet of follicular cells with internal microfollicles with low-grade nuclear features of malignancy (40X, MGG)

The smear showed multiple monolayered cell sheets as well as cell sheets with the globoid shape of thyroid follicular cells with internal microfollicles. Also seen are isolated microfollicles of dissociated follicular cells. Cells are cuboidal in shape and carry hyperchromatic nuclei with mild pleomorphism, granular uneven chromatin and frequent nucleoli. The cytoplasm is modest. These cells are seen to be entrapped within native stroma. Background showed hemorrhagic material, sparse lymphocytes and isolated stromal fragments (Figure 2, Figure 3). The diagnosis of deposits of Thyroid Follicular Carcinoma was suggested without differential diagnosis. The USG examination for thyroid was suggested in the report on cytopathology. The clinician upon the cytopathology report of the scalp FNA sent patient for evaluation for Contrast-Enhanced Computed Tomography (CECT) neck with thorax. The CECT neck showed heterogeneously enhancing soft tissue density lesion in the right lobe of thyroid placed with intraparenchymal tissue location. The swelling was placed deeply with a size of 9 x 6.5 x 6 cm, which was causing deviation of trachea, esophagus and larynx towards the left. The lesion within the right lobe of thyroid was seen in the deep right common carotid artery, causing its lateral displacement. The CECT neck and thoracoabdomen also showed the lymph node on the right side of small size showing heterogenous enhancement. The left lobe of the thyroid was reported to be normal.

**Figure 4:**
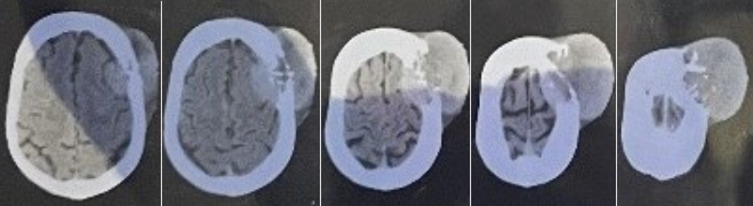
computer tomography scan of head showing soft tissue swelling eroding partial bone underneath

The patient was also reported to have a lytic lesion in the body of T10 vertebrae. A few small yet multiple heterogeneously enhancing soft tissue densities were reported in the bilateral lung parenchyma in the basal segment of the left lower lobe. The CECT also reported the characteristic of mass on the scalp which on FNAC was reported as metastasis of Thyroid Follicular Carcinoma as below; size 6.5 x 6 x 3 cm, punctuate calcification, bony erosion of frontal and parietal bone, no brain involvement was appreciated. The diagnosis offered for the lesion on the scalp was of metastatic nodule (Figure 4). The liver showed multiple metastatic lesions in segments III, IV, V, VI & VIII. The pole of the left kidney was reported to carry a simple cortical cyst. The lobe underwent USG-guided FNAC of deeply located thyroid lesions by standard methods. The smears of thyroid aspirate were stained by May-Grunwald-Giemsa and Papanicolaou stain.

**Figure 5:**
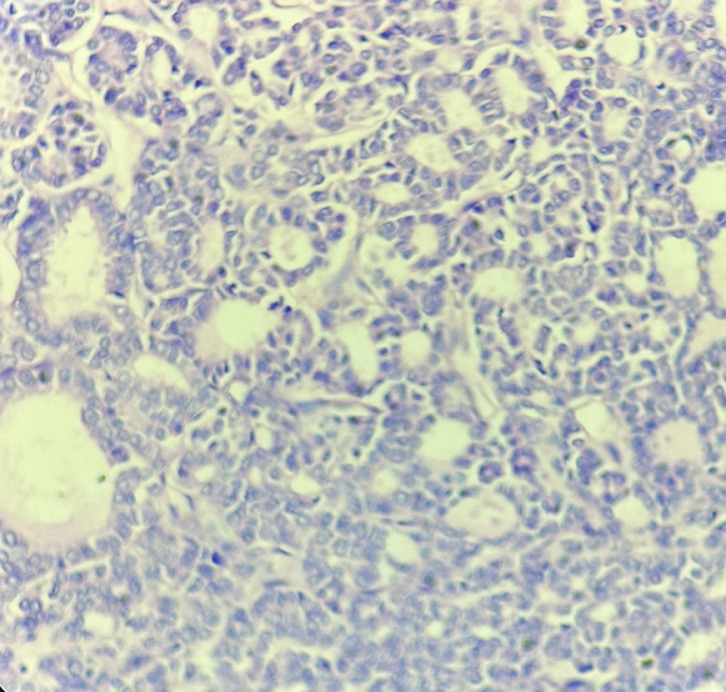
photomicrograph, metastatic follicular thyroid carcinoma thyroid: section showing malignant thyroid follicles accompanied by solid growth pattern (HP, 40X, HE)

**Figure 6:**
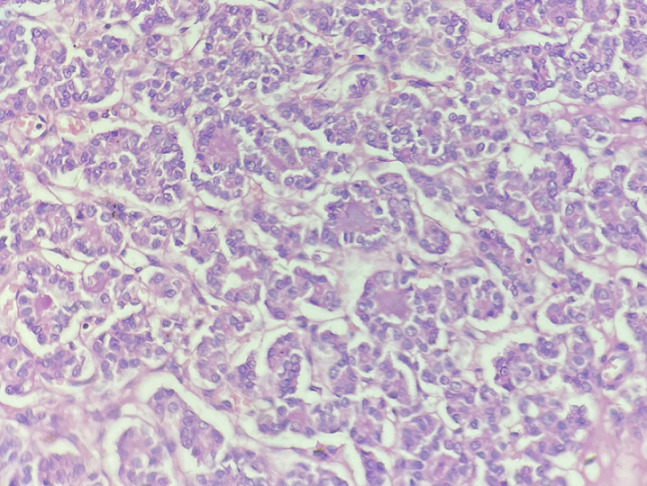
photomicrograph, primary follicular thyroid carcinoma scalp: section showing malignant thyroid follicles and malignant follicular cells (HP, 40X, HE)

The smear showed multiple sheets of follicular cells with internal microfollicles. Also seen in smears are multiple microfollicles and a few isolated follicular cells. The nuclei of follicular cells show hyperchromasia and frequent nucleation. A few cells show mild nuclear pleomorphism and uneven chromatin. The cytoplasm of the cells is cuboidal and granular in a few. The background shows reduced bare nuclei and a rare small flake of thick colloid. Also seen in smears are thyroid stromal cells of thin and thick character entrapping microfollicles. The background is generally hemorrhagic. The cytopathological diagnosis of follicular neoplasm favoring follicular carcinoma was made (TBS -5). The patient with cytopathology report of follicular neoplasm favoring follicular carcinoma was advised to undergo a total thyroidectomy by the surgeon. The patient underwent thyroidectomy for both thyroid and metastatic lesions on the scalp (Figure 5, Figure 6). The histopathological diagnosis from an excised tumor on the scalp was diagnosed as metastasis of follicular thyroid carcinoma. The IHC for thyroid transcription factor 1 (TTF1) was carried out on the section of the tumor of the thyroid and excised tumor from the scalp and was positive for it.

### Diagnosis

The cytopathological diagnosis of follicular neoplasm favoring follicular carcinoma was made (TBS -5).

### Therapeutic interventions

The patient underwent thyroidectomy for both thyroid and excision of metastatic lesions on the scalp.

### Follow-up and outcome of interventions

The patient is receiving routine follow-up and there is no recurrence at 1-year post-surgery.

### Patient perspective

The course of therapy and the patient's level of recovery pleased the patient.

### Informed consent

The patient granted their written, informed consent for the case report and any related images to be published.

## Discussion

The cytodiagnosis of metastasis of follicular thyroid carcinoma to the scalp and skull has been reported rarely. Mukherjee *et al*. [1], Koppad *et al*. [3] and Yang *et al*. [5] have reported FNA diagnosis of metastasis of follicular thyroid carcinoma, where the thyroid on the clinical examination was normal. FNA diagnosis of metastatic lesion of follicular thyroid carcinoma has been reported by Mukherjee *et al*. at scalp [1]. FNA diagnosis of metastatic lesion of follicular thyroid carcinoma has been reported by Koppad *et al*. [3] on skull. FNA diagnosis of metastatic lesion of follicular thyroid carcinoma has been reported by Yang *et al*. on skull base [5]. Such clinical occult primary follicular thyroid carcinoma metastasizing to the scalp and distant organs is a rare biological phenomenon.

The metastatic lesion of follicular thyroid carcinoma to the scalp in the absence of the thyroid swelling mimics a benign scalp tumor. Therefore, the cytomorphological interpretation from a material of FNAC from this tumor is dicey. However, the familiarity of cytomorphological patterns of primary follicular thyroid carcinoma which is identified by the presence of cuboidal malignant cells placed in microfollicular in plenty. The other features that help in the diagnosis of follicular thyroid carcinoma metastasis or the absence of bare nuclei, well differentiated morphology and rudimentary presence of colloid in the aspirated material. There are reports on the diagnosis of metastatic lesions of follicular thyroid carcinoma by FNA in patients known primarily to have follicular thyroid carcinoma.

Bhansali *et al*. [6] reported pulsatile osseous metastasis of follicular thyroid carcinoma on FNAC, while Gandhoke *et al*. [7] reported massive skull metastases of follicular thyroid carcinoma by FNAC. Mukherjee *et al*. [1] reported cytodiagnosis of scalp metastasis of follicular thyroid carcinoma.

There are reports by Meena *et al*. [2], Gupta *et al*. [4], Bhansali *et al*. [6], Agarwal *et al*. [8], Kumar *et al*. [9], and Starkar *et al*. [10] where in the metastatic lesion of follicular thyroid carcinoma has been reported on FNAC in patients with known primary follicular thyroid carcinoma.

The cytomorphology of metastatic lesions of follicular thyroid carcinoma is similar to one of the primary lesions of follicular thyroid carcinoma in the thyroid. This observation of ours has been endorsed in the case reports of above authors.

The clinical examination of the present case revealed no clinically appreciable primary tumor. It was discovered on examination of sonography and CECT of neck after FNA report of metastatic lesion of follicular thyroid carcinoma in the scalp. Such observation has not been entertained in the studies reviewed for the present one.

## Conclusion

FNAC of bumpy lesion on the scalp is useful enough in differentiating between a primary scalp tumor versus the metastatic lesions. Furthermore, FNA diagnosis of follicular thyroid carcinoma may prompt clinicians to search for primary within the thyroid which may otherwise remain clinically silent. This is more true with follicular thyroid carcinoma as it is biologically aggressive and widely metastatic.
